# Association of air pollution and use of glyceryl trinitrate against angina pectoris: a population-based case-crossover study

**DOI:** 10.1186/1476-069X-12-38

**Published:** 2013-04-30

**Authors:** Ragnhildur Gudrun Finnbjornsdottir, Helga Zoëga, Orn Olafsson, Throstur Thorsteinsson, Vilhjalmur Rafnsson

**Affiliations:** 1 Center of Public Health Sciences, Faculty of Medicine, University of Iceland, Reykjavík, Iceland; 2 Environment and Natural Resources, University of Iceland, Reykjavík, Iceland; 3 Institute of Earth Sciences, University of Iceland, Reykjavík, Iceland; 4 Department of Preventive Medicine, University of Iceland, Reykjavík, Iceland

**Keywords:** Air pollution, Angina pectoris, Coronary disease, Case-crossover, Drug utilization, Nitrogen dioxide, Ozone, Pharmacoepidemiology

## Abstract

**Background:**

Ambient air pollution has been associated with increased cardiovascular morbidity and mortality. In Reykjavik, Iceland, air pollutant concentrations exceed official health limits several times every year. The aim was to study the association of concentrations of NO_2_, O_3_, PM_10_, and H_2_S in the Reykjavik capital area with the dispensing of anti-angina pectoris medication, *glyceryl trinitrate* to the inhabitants.

**Methods:**

Data on daily dispensing of *glyceryl trinitrate*, were retrieved from the Icelandic Medicines Registry. Data on hourly concentrations of NO_2_, O_3_, PM_10_, and H_2_S were obtained from the Environment Agency of Iceland. A case-crossover design was used, based on the dispensing of *glyceryl trinitrate* to 5,246 individuals (≥18 years) between 2005 and 2009.

**Results:**

For every 10 μg/m^3^ increase of NO_2_ and O_3_ 3-day mean concentrations, the odds ratio (OR) for daily dispensing of *glyceryl trinitrates* was 1.136 (95% confidence intervals (CI) 1.069-1.207) and 1.094 (95% CI 1.029-1.163) at lag 0, and OR was 1.096 (95% CI 1.029-1.168) and 1.094 (95% CI 1.028-1.166) at lag 1, respectively.

**Conclusions:**

These findings suggest that NO_2_ and O_3_ ambient air concentrations may adversely affect cardiovascular health, as measured by the dispensing of *glyceryl trinitrates* for angina pectoris. Further, the findings suggest that data on the dispensing of medication may be a valuable health indicator when studying the effect of air pollution on cardiovascular morbidity.

## Background

In previous studies an increased risk of cardiovascular morbidity and mortality has been found in association with exposure to air pollution [1-3]. In these studies different outcome measures were used such as hospital admission [4], emergency department visits [5], or deaths [6,7] due to cardiovascular diseases. On the other hand, some studies do not show an association between the risk of cardiovascular morbidity and mortality and air pollution [8,9].

Zeghnoun and coworkers [10] introduced the dispensing of respiratory drugs as a novel indicator to measure respiratory morbidity in association with air pollution. They implied that drug dispensing generally reflects less severe morbidity states than those resulting in emergency department visits, hospitalizations or death, and could therefore serve as a sensitive marker to measure the effects of air pollution in small populations where mortality rates are low. Since then, only a few studies have investigated the association of air pollution and the dispensing of anti-asthma drugs as an indicator of respiratory symptoms or asthma attacks [11-14].

To our knowledge, the dispensing of medication for cardiovascular diseases has not previously been used to estimate the possible effect of ambient air pollution on cardiovascular health. In the following study, the aims were to evaluate the association of changes in ambient air concentrations of NO_2_, O_3_, PM_10_, and H_2_S with dispensing of the sublingual medication *glyceryl trinitrate* used against attacks of angina pectoris.

## Methods

**Figure 1:**
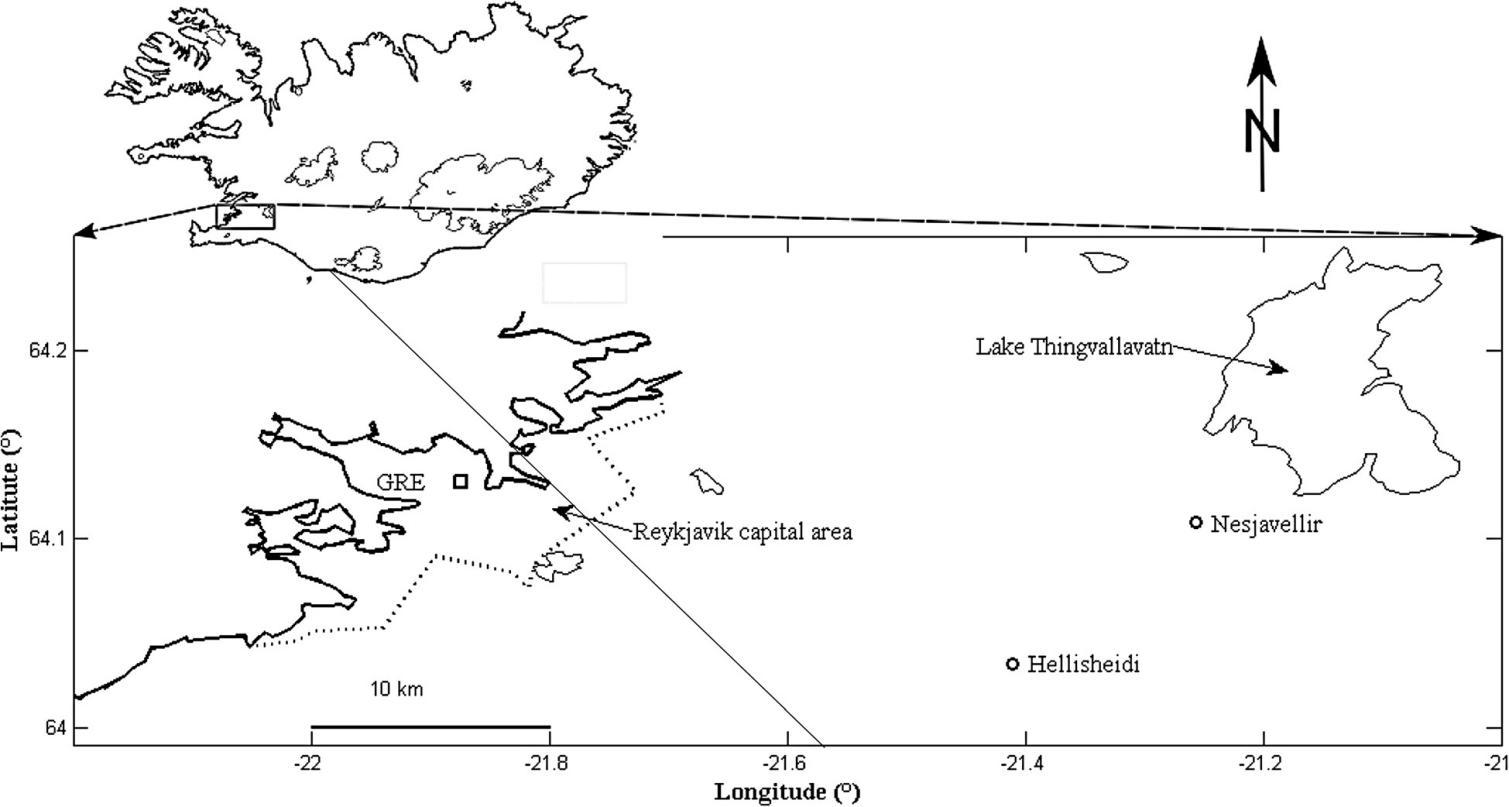
**Map of the location of NO_2_, O_3_, PM_10_, and H_2_S measurement device (GRE).** The measurement location is by the Grensasvegur-Miklabraut intersection in the Reykjavik capital area of Iceland. Dotted line indicates the boundary of the capital area and circles indicate point sources of H_2_S emissions, the Hellisheidarvirkjun and Nesjavallavirkjun power plants.

The study base consisted of inhabitants 18 years or older in the Reykjavik capital area (communities: Reykjavik, Seltjarnarnes, Kopavogur, Hafnarfjordur, Gardabaer, and Mosfellsbaer) identified by postal codes (Figure 1). According to Statistics Iceland the annual mean population was 142,789 individuals (≥18 years) [15]. The study period was January 1, 2005 to December 31, 2009.

### Exposure and covariate assessment

The Environment Agency of Iceland provided the exposure data. The agency continuously measures air pollution levels of various pollutants at a busy intersection of two main roads in Reykjavik (Grensasvegur-Miklabraut) where on average around 60,000 cars cross daily (Figure 1) [16]. In this study, 1-hour measurements were obtained for NO_2_, O_3_, PM_10_, and H_2_S concentrations. Gaps in exposure measurements were due to occasionally inactive measuring equipment. PM_10_ was measured with an Andersen EMS IR Thermo (model FH62 I – R, Thermo Scientific, United States) and NO_2_, H_2_S, and O_3_ with a Horiba (models APNA 360E, APSA 360ACE, and APOA 360E, Horiba, United States). These devices are calibrated every six to twelve months.

The entire dataset contained 1,826 days (5 years). The 24-hour mean concentration values (from the time 00:00 to 00:00 the following day) were calculated for each pollutant, using the 1-hour values where at least 75% of the data existed. The number of days in which the 75% criterion was fulfilled varied by pollutant; missing 24-hour values were 143 (8%) for NO_2_, 83 (4%) for O_3_, and 36 (2%) for PM_10_. H_2_S measurements started on February 22, 2006 and therefore do not provide complete data. H_2_S measurements thus contribute 1,409 days to the dataset, for which 219 days (15%) were missing. The NO_2_, O_3_, and PM_10_ are traffic related, however deserts dust and volcanic ashes contribute to the concentration of PM_10_ in the Reykjavik area depending on wind direction. The source of H_2_S is two geothermal power plants 26 and 33 km east of the city. For meteorological data, 24-hour mean values were retrieved from the same measurement location as for the pollutants. Missing days were 13 (<1%) for temperature and relative humidity (RH). Missing values were not associated with any particular day in the week. The 3-day means were calculated for each pollutant and meteorological data based on 24-hour values, giving the running averages of the day when dispensing occurred (index day), one day before and two days before the index day, so the 3-day means included the day of dispensing.

Monthly numbers of influenza cases were obtained from the Directorate of Health and were recalculated into binary variables with a cut-off of 300 cases per month to define an influenza season.

### Case ascertainment

The Icelandic Medicines Registry was the source of the outcome data and the registry contains information for each person to whom prescription medication was dispensed as an outpatient during the study period. The coverage of the database is virtually complete in the Reykjavik area for all filled prescriptions in outpatient settings [17]. For each occurrence of *glyceryl trinitrate* dispensing in the study we received information on medication name, number of defined daily doses (DDDs), Anatomic Therapeutic Chemical (ATC) code [18], dispensing date, encrypted personal identification number, sex, birth year, and residential postal code of the patient. The personal identification number allowed checking whether drug dispensing had occurred with seven days intervals, to avoid overlap with control days.

We retrieved information on all filled prescriptions defined as *glyceryl trinitrates* (ATC code: C01DA02), the target medication in this study. *Glyceryl trinitrates* are used against an abrupt onset of painful attacks of angina pectoris. An effective dose of *glyceryl trinitrate* administered sublingually acts within minutes, usually terminating pain quickly and completely, making it an ideal medication to study in association with air pollution fluctuations. No information on underlying medical conditions was available from the Icelandic Medicines Registry. Other drugs used against angina pectoris were not taken into consideration in this study. These are long acting nitrates, beta blockers, and calcium channel blockers and when used against angina pectoris these medicines should be taken regularly. Beta blockers and calcium channel blockers are indicated for several other medical conditions than angina pectoris. The *glyceryl trinitrate* is the only medicine against angina pectoris used intermittently when the patient has an attack of chest pain. In the study all drug dispensing was according to prescription and it was not possible to differ between new prescription and renewal fills due to routine or exacerbation of symptoms. Some patients may have had prescription valid for up to four repeated fillings, and recognition of these prescriptions was not possible. A package of *glyceryl trinitrate,* which has been opened expires in eight weeks.

Three preconditions were needed to be defined as a case; 1) the individual was living in the Reykjavik capital area, 2) the individual was 18 years or older, and 3) *glyceryl trinitrates* were dispensed to the individual at least once over the study period. The total number of cases over the study period was 5,246.

### Design and data analysis

Pearson’s correlation was used to assess the inter-correlation between exposure factors and the meteorological covariates (Table 1).

**Table 1:** Air pollution statistics

	**Days, n**	**Mean (SD)**	**Min**	**Max**	**Pearson's correlation coefficient**					
					**PM_10_**	**NO_2_**	**O_3_**	**H_2_S**	**Rel. humidity, %**	**Temperature, °C**
**24-hour mean**										
PM_10_, μg/m^3^	1,790	22.6 (20.8)	3.2	261.6	1					
NO_2_, μg/m3	1,683	20.8 (13.5)	0.7	100.6	0.13	1				
O_3_, μg/m^3^	1,744	40.7 (13.8)	1.2	144.5	0.13	−0.62	1			
H_2_S, μg/m^3^	1,190	3.8 (6.7)	0.0	92.5	0.02	0.34	−0.28	1		
Rel. humidity, %	1,813	77.8 (10.9)	39.3	102.5 ^a^	−0.33	0.04	−0.07	0.03	1	
Temperature, °C	1,813	5.9 (5.2)	−10.3	22.2	−0.28	−0.43	−0.04	−0.28	0.15	1
**3-day mean**					**PM_10_**	**NO_2_**	**O_3_**	**H_2_S**	**Rel. humidity, %**	**Temperature, °C**
PM_10_, μg/m^3^	1,811	20.5 (15.3)	4.6	125.7	1					
NO_2_, μg/m^3^	1,707	20.7 (10.9)	0.7	100.6	0.11	1				
O_3_, μg/m^3^	1,765	40.8 (11.5)	5.8	108.9	0.22	−0.52	1			
H_2_S, μg/m^3^	1,282	3.8 (5.1)	0.1	69.5	−0.01	0.41	−0.29	1		
Rel. humidity, %	1,824	77.8 (9.1)	43.0	101.2^a^	−0.34	0.03	−0.07	0.05	1	
Temperature, °C	1,824	5.9 (5.0)	−7.3	18.2	−0.43	−0.48	−0.12	−0.34	0.14	1

Abbreviations: H_2_S: hydrogen sulfide; Max: maximum value over specific time period; Mean: mean value over specified time period; Min: minimum value over specific time period; NO_2_: nitrogen dioxide; O_3_: ozone; PM_10_: particulate matter with an aerodynamic diameter of ≤10 μm; Rel. humidity: relative humidity; SD: standard deviation.

^a^ The maximum value of relative humidity is 100%. There is a 2.5% error margin in the measuring equipment.

Description of air pollution in Reykjavik, Iceland, over the study period of January 1^st^ 2005 to December 31^st^ 2009. 24-hour mean values and 3-day mean values are shown as well as Pearson’s correlation coefficient between pollutant factors.

A case-crossover design was used, as it is suitable for studying the association of transient exposure, such as air pollution, and an acute onset event, such as the dispensing of anti-angina pectoris medication, *glyceryl trinitrate*[19]. The symmetric bidirectional design with selection of two controls, seven days before and after the index day (day of dispensing *glyceryl trinitrate)* was used [20]. The case-crossover design compares each person’s exposure experience in a time period just prior to the case-defining event with that same person’s exposure experience seven days before and seven days after the event. This matches controls for measured or unmeasured personal confounding characteristics that do not vary over the relatively short time, such as gender, age, and genetic factors [20].

We conducted several calculations including single pollutants NO_2_, O_3_, PM_10_, H_2_S, temperature, humidity, and influenza seasons to each single pollutant model. Two separate multi pollutant models were used, one introducing three pollutants, NO_2_, O_3_, and PM_10,_ based on 24-hour means of the pollutants, and another introducing four pollutants, NO_2_, O_3_, PM_10_, and H_2_S both adjusted for temperature and humidity on the same day. Finally, all models were also run with pollutant exposure averaged over three consecutive days (3-day means), also adjusted for temperature and humidity. These models were also run with and without adjustment for the influenza periods.

A lag time up to three days was introduced in the analyses according to the following definitions: lag 0: air pollution exposure on the dispensing day, lag 1-3: air pollution exposure one day before (lag 1) and up to three days before (lag 3) the dispensing day. We obtained estimates of odds ratios (OR) for the association of each pollutant, using conditional logistic regression. Sets were excluded if there were two or more missing values in the matched case control set (a complete set contains three exposure values). We reported effects estimates as the change in dispensing of *glyceryl trinitrate* associated with 10 μg/m^3^ increase in 24-hour mean or 3-day mean pollutant levels.

A few conditional logistic regression calculations with different controls in the symmetric bidirectional case-crossover design were conducted. Firstly, both days of the bidirectional controls were used, i.e., the same day of the week one week before and one week after the index day; secondly, one control day was used, the same day of the week one week before the index day; thirdly, one control day was used, the same day of the week one week after the index day; and fourthly, one control day was randomly chosen from the same day of the week either a week before or a week after the index day. This fourth procedure is called semisymmetric bidirectional control selection [20]. If only one of these days was available, as a result of the case being at either the end of the exposure series, that day served as the sole control day. Overlap bias is avoided by the semisymmetric bidirectional design [20]. All these calculations gave nearly identical results and therefore the only results based on the symmetric bidirectional controls are shown in this presentation.

We used SPSS 16.0 (IBM software, United States) and STATA 11 (StataCorp, United States) for all analyses. The study protocol was approved by the National Bioethics Committee (ref. no. VSNb2010030008/03.7) and the Data Protection Authority (ref. no. 2010030105/5.2.3.3/SH/sh).

## Results

### Descriptive statistics

Excluding repeated dispensing occurrences to the same patient on the same day, *glyceryl trinitrates* were dispensed 8,604 times to a total of 5,246 individuals (57.9% males, 40.1% females) over the study period. On average, dispensing of *glyceryl trinitrate* occurred 4-5 times each day to the patients in the study base, range 0 to 19 per day. The mean age of the patients was 74 years (median 76 years, interquartile range 67, 82 years). Around 98% of individuals who used *glyceryl trinitrates* filled fewer than five prescriptions per year, and approximately 73% filled one prescription per year. The majority of dispensing occurrences took place on weekdays rather than on weekends or holidays.

**Figure 2:**
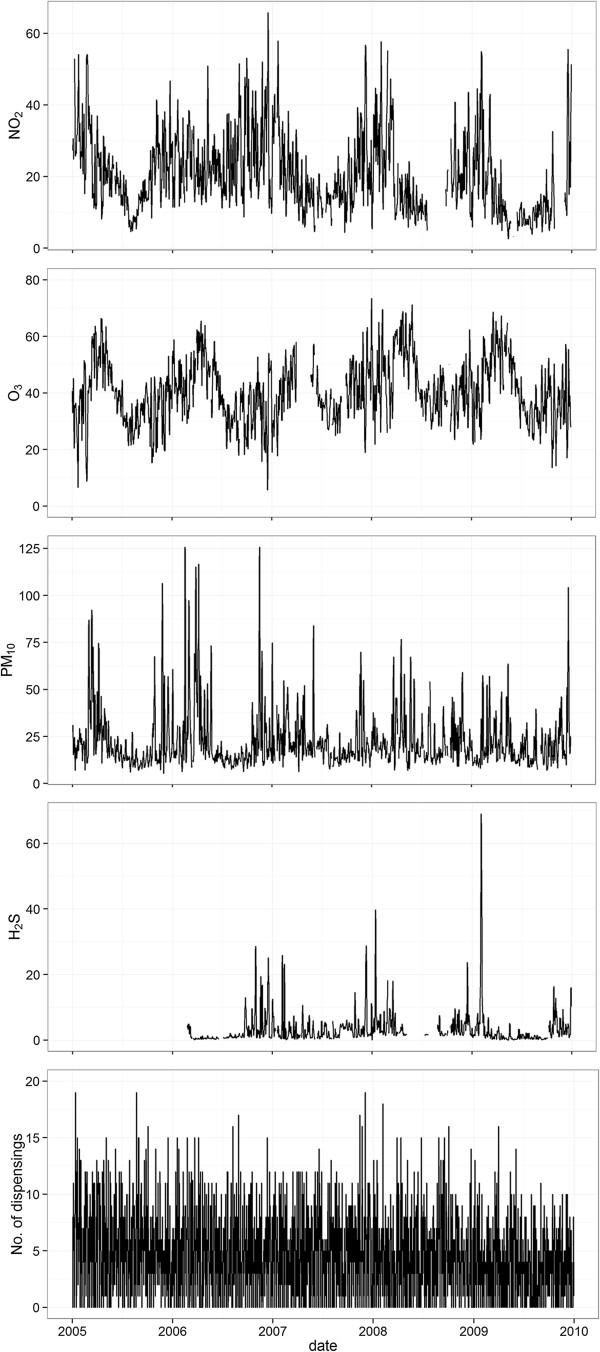
**Time-series plot of air pollutants and *glyceryl trinitrate *drug dispensing.** 3-day mean concentration of pollutants (μg/m^3^) and daily number of *glyceryl trinitrate* drug dispensing in Reykjavik, Iceland over the study period of January 1, 2005 to December 31, 2009. Measurements of H_2_S started in February 2006. Gaps in figures are due to missing data.

NO_2_ and O_3_ followed a seasonal pattern with higher concentrations during winter, whereas such a pattern was not as clear for PM_10_ and H_2_S (Figure 2). The 24-hour mean during winter months (November 1 to April 30) was 25 μg/m^3^ (standard deviation (SD) 15) and 43 μg/m^3^ (SD 15) for NO_2_ and O_3_, respectively. Over the summer months (May 1 to October 31) the 24-hour mean was 16 μg/m^3^ (SD 15) and 38 μg/m^3^ (SD 12) for the same pollutants. The inter-correlation was strongest between 24-hour mean concentration levels of NO_2_ and O_3_ where there was a negative correlation of -0.62 (Table 1), while the strongest positive correlation of 0.41 was between the 3-day means of NO_2_ and H_2_S. Temperature was negatively correlated with each pollutant and relative humidity correlated negatively with PM_10_ and O_3_ (Table 1).

### Air pollution and *glyceryl trinitrate* dispensing

The matched OR and 95% CI for the dispensing of *glyceryl trinitrate* associated with every 10 μg/m^3^ increase in NO_2,_ O_3,_ PM_10,_ and H_2_S 24-hour mean, as well as temperature, humidity, and influenza season in single pollutant model, applying lag 0 and 1 can be seen in Table 2. A significant association was found between the dispensing of *glyceryl trinitrate* and the 24-hour mean concentrations of NO_2_ with lag 0. Due to the low correlation between pollutants (Table 1), and the complex exposure to many measured pollutants, a multipollutant model was also applied (Table 3). A significant positive association was found between the dispensing of *glyceryl trinitrate* and the 24-hour mean concentrations of NO_2_ and O_3_. At lag 0 and 1 a significant odds ratio (OR) of 1.059 (95% CI: 1.012, 1.108) and 1.074 (95% CI: 1.025, 1.126) for NO_2_ was evident. For O_3,_ there was a significant OR of 1.087 (95% CI: 1.035, 1.141) at lag 1. The multi-pollutant model was run with and without H_2_S and when introducing H_2_S in the model, the pattern of the association of the other exposure factors did not change. The 95% CI of the ORs for 24-hour mean concentrations of H_2_S and PM_10_ include unity at every time lag and are therefore not statistically significant (Table 3).

**Table 2:** **Association of 24-hour air pollutant concentrations and *glyceryl trinitrate *dispensing in a single pollutant model**

	**NO_2_**		**O_3_**		**PM_10_**		**H_2_S**	
**Lag**	**OR**	**95% CI**	**OR**	**95% CI**	**OR**	**95% CI**	**OR**	**95% CI**
**0**	1.038	1.011, 1.062	0.988	0.965, 1.012	1.005	0.929, 1.017	0.997	0.991, 1.004
**1**	1.015	0.991, 1.040	1.017	0.993, 1.041	1.005	0.992, 1.018	0.937	0.874, 1.000
	**Temperature**		**Relative humidity**		**Influenza seasons**			
**Lag**	**OR**	**95% CI**	**OR**	**95% CI**	**OR**	**95% CI**		
**0**	1.019	0.936, 1.111	1.020	0.994, 1.046	4.300	0.721, 25.626		
**1**	0.986	0.904, 1.074	1.001	0.976, 1.027	1.976	0.336, 11.634		

The matched odds ratios (OR) and 95% confidence intervals (CI) for the dispensation of *glyceryl trinitrate* in Reykjavik, Iceland, associated with every 10 μg/m^3^ increase in NO_2_, O_3_, PM_10_, H_2_S, temperature, and relative humidity 24-hour mean concentrations, and influenza seasons.

**Table 3:** **Association of air pollutants and *glyceryl trinitrate *dispensing in a multi-pollutant model**

	**NO_2_**		**O_3_**		**PM_10_**		**H_2_S**	
**Lag**	**OR**	**95% CI**	**OR**	**95% CI**	**OR**	**95% CI**	**OR**	**95% CI**
**24-hour mean**								
**Model not including H**_**2**_**S**								
**0**	1.069	1.028, 1.112	1.036	0.995, 1.078	1.005	0.991, 1.018		
**1**	1.081	1.038, 1.125	1.088	1.045, 1.133	0.999	0.985, 1.014		
**2**	1.037	0.994, 1.083	1.036	0.995, 1.080	1.003	0.988, 1.019		
**3**	1.027	0.983, 1.074	1.020	0.978, 1.064	0.988	0.971, 1.004		
**Model including H**_**2**_**S**								
**0**	1.059	1.012, 1.108	1.015	0.968, 1.065	1.002	0.985, 1.020	0.987	0.933, 1.045
**1**	1.074	1.025, 1.126	1.087	1.035, 1.141	0.995	0.976, 1.013	0.958	0.907, 1.013
**2**	1.054	1.003, 1.108	1.047	0.996, 1.101	1.001	0.982, 1.020	0.972	0.922, 1.025
**3**	1.032	0.980, 1.087	1.028	0.976, 1.083	0.983	0.962, 1.004	1.030	0.979, 1.083
**3 day mean**								
**Model not including H**_**2**_**S**								
**0**	1.116	1.059, 1.176	1.090	1.036, 1.146	1.009	0.988, 1.030		
**1**	1.071	1.014, 1.130	1.072	1.019, 1.129	0.998	0.976, 1.021		
**2**	1.013	0.957, 1.071	1.018	0.965, 1.073	0.996	0.973, 1.019		
**3**	0.977	0.923, 1.034	0.990	0.938, 1.046	1.000	0.978, 1.022		
**Model including H**_**2**_**S**								
**0**	1.136	1.069, 1.207	1.094	1.029, 1.163	0.998	0.972, 1.024	0.934	0.866, 1.008
**1**	1.096	1.029, 1.168	1.094	1.028, 1.166	0.988	0.961, 1.015	0.975	0.906, 1.048
**2**	1.045	0.978, 1.117	1.038	0.972, 1.108	0.983	0.956, 1.012	1.003	0.933, 1.079
**3**	0.993	0.929, 1.061	0.997	0.933, 1.065	0.985	0.958, 1.013	1.027	0.951, 1.109

Data have been calculated in a unique multivariate analysis separately for each lag, taking into account simultaneously each pollutant variables.

No. of observations for 24-hour mean not including H_2_S: lag 0: 22,436; lag 1: 22,327; lag 2: 22,288; and lag 3:22,367. No. of observations for 24-hour mean including H_2_S: lag 0: 13,816; lag 1: 13,686; lag 2: 13,598; and lag 3: 13,650. No. of observations for 3-day mean not including H_2_S: lag 0: 21,913; lag 1: 21,755; lag 2: 21,617; and lag 3: 21,682. No. of observations for 3-day mean including H_2_S: lag 0: 13,816; lag 1: 13,686; lag 2: 13,598; and lag 3: 13,650.

The matched odds ratios (OR) and 95% confidence intervals (CI) for the dispensation of *glyceryl trinitrate* in Reykjavik, Iceland, associated with every 10 μg/m^3^ increase in NO_2_, O_3_, PM_10_, and H_2_S 24-hour mean and 3-day mean concentrations. Adjusted for each pollutant variable, temperature, and relative humidity.

**Figure 3:**
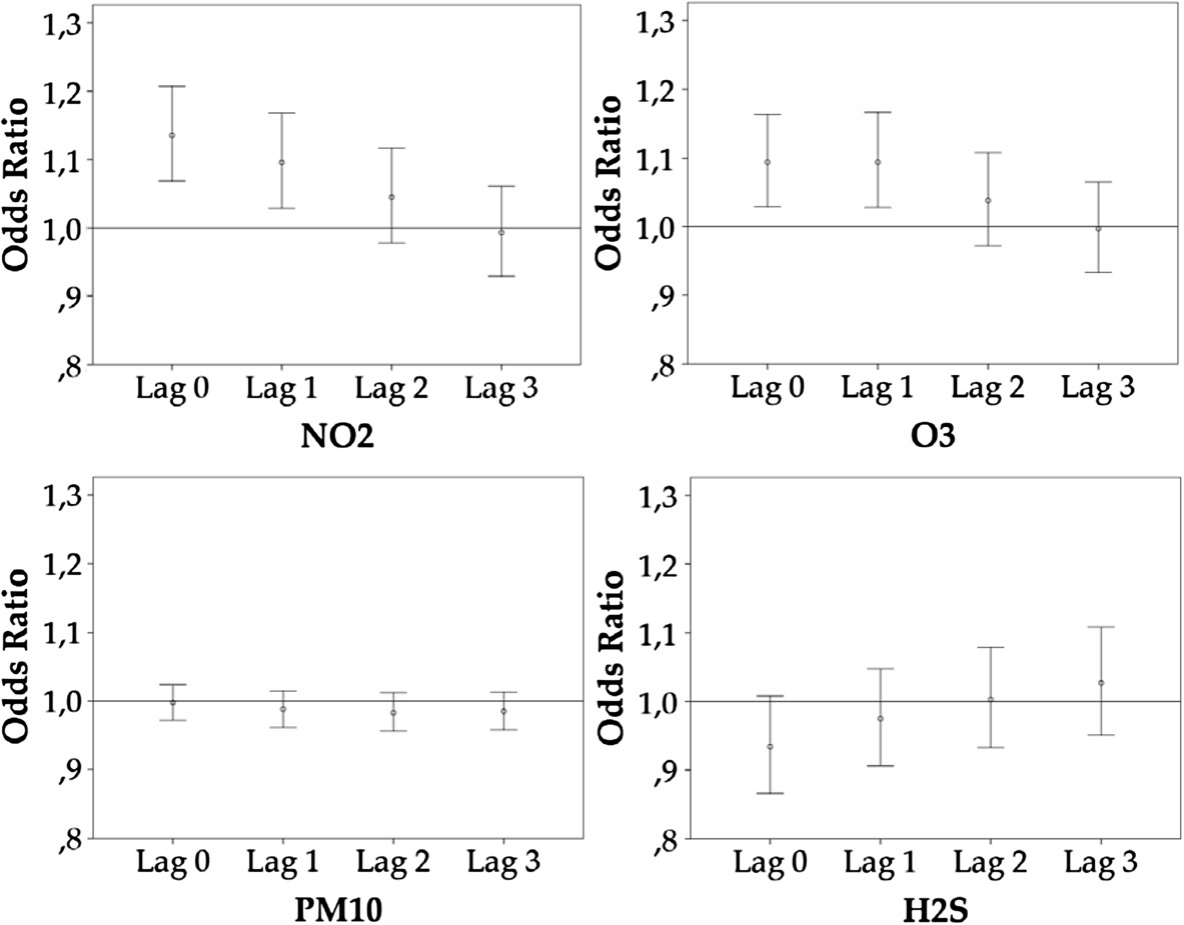
**Association of NO_2_, O_3_, PM_10_, and H_2_S exposure (3-day mean) and daily *glyceryl trinitrate *dispensing.** Figure shows up to lag 0-3 days, adjusted for each pollutant variable, temperature, and relative humidity. Bars show 95% confidence intervals (CI).

The 3-day mean concentration values yielded a stronger association than the 24-hour mean concentration values. At lag 0 the OR was 1.136 (95% CI: 1.069, 1.207) and 1.094 (95% CI: 1.029, 1.163) in daily occurrences of *glyceryl trinitrate* dispensing following an increase in the 3-day mean concentrations of NO_2_ and O_3_, respectively. At lag 1, the increase in occurrences of *glyceryl trinitrate* dispensing was 1.096 for NO_2_ (95% CI: 1.029, 1.168) and 1.094 for O_3_ (95% CI: 1.028, 1.166). The OR at lag 2 and 3 was not statistically significant for either pollutant (Table 3 and Figure 3). H_2_S showed a different pattern compared with NO_2_ and O_3,_ where lag 0 and lag 1 showed a decrease (lag 0: 0.934; 95% CI: 0.866, 1.008 and lag 1: 0.975; 95% CI: 0.906, 1.048). At lag 2 and lag 3 there was an increase of 1.003 (95% CI: 0.933, 1.079) and 1.027 (95% CI: 0.951, 1.109), respectively. ORs for H_2_S were not statistically significant at any time lag. PM_10_ also had a different pattern than the other pollutants where the OR was around 1 at every time lag and therefore not statistically significant at any time lag (Table 3 and Figure 3).

Introducing the influenza season to the models did not change the pattern of ORs for these exposures, as the ORs became only slightly elevated when the influenza season was included. The OR for influenza season was in no instance statistically significant as a confounder on a 5% level, except in the model when H_2_S was not included and at lag 0.

## Discussion

In this study we found that exposure to urban air pollution was associated with the dispensing of *glyceryl trinitrates* (ATC code: C01DA02) among adults living in the Reykjavik area. The results show an increase of 13.6% and 9.6% in occurrences of *glyceryl trinitrate* dispensing for every 10 μg/m^3^ increase in the 3-day mean of NO_2_ at lag 0 and lag 1. Likewise, an increase of 9.4% and 9.4% in *glyceryl trinitrate* dispensing for every 10 μg/m^3^ increase the 3-day mean of O_3_ at lag 0 and lag 1 was evident.

Air pollution is believed to affect the biological pathway of the cardiovascular mechanism [21-23] but in what way is not yet clear. An experimental study on humans indicated that inhalation of fine particulate air pollution and ozone may cause acute arterial vasoconstriction that could lead to cardiovascular events [24]. A similar study showed that air pollution impairs endothelial function in young healthy males [25].

This study found an increase in dispensing of *glyceryl trinitrate* on the same day as increases NO_2_ and O_3_ occurred (lag 0). When an increase in NO_2_ and O_3_ occurred the day before (lag 1), there was also an increase in *glyceryl trinitrate* dispensing. This is evident for both 24-hour mean and 3-day mean concentrations. One of the major conditions for concluding on causality, that the exposure precedes the outcome, is fulfilled in the present study [26]. The 3-day mean of NO_2_ and O_3_ that is partly occurring before the index day gave higher ORs than the 24-hour mean concentrations, and thus a stronger association with the dispensing of *glyceryl trinitrate*.

Using the 3-day mean eliminates the possibility that the dispensing occurs before the exposure, whereas when using the 24-hour mean, the dispensing could theoretically occur during the exposure, as the 3-day mean yielded higher OR than the 24-hour mean. We also found a strong statistically significant increase in dispensing of *glyceryl trinitrate* at lag 1 for NO_2_ and O_3_, suggesting that patients use more *glyceryl trinitrates* when increases in the 3-day mean occur one day before the index day.

Concurrent with our results, Bhaskaran and coworkers [27] found that 10 μg/m^3^ increases in NO_2_ levels were associated with a 1.1% increased risk of myocardial infarction (MI) only 1-6 hours after the exposure occurred, a time frame that would have fallen within lag 0 in our study. However, we did not find an association between PM_10_ and *glyceryl trinitrate* dispensing, while Bhaskaran and coworkers demonstrated a 1.2% increase of MI with every 10 μg/m^3^ increase in PM_10_[27]. Moreover, the Bhaskaran study is inconsistent with our results, in that they found no association between O_3_ and MI. Quite a few studies have demonstrated elevated relative risks, measured as MI [28,29], hospital admissions [30], and emergency department visits [31] due to cardiovascular diseases, on the same day and one day after increases in NO_2_ and O_3_ concentration occur (lag 0 and 1) [28,30]. Notably, von Klot and coworkers found a 1.32% increase in readmissions due to angina pectoris, the condition for which *glyceryl trinitrates* is prescribed, following an 8 μg/m^3^ increase in NO_2_ concentrations [30].

PM_10_ was not associated with *glyceryl trinitrate* dispensing at any lag in our study. Previous case-crossover studies have demonstrated an association of PM_10_ and PM_2.5_ with cardiovascular morbidity [4,29,32]. Some other studies have failed to find a statistically significant association of PM and cardiovascular morbidity [8,9]. Our findings concerning PM_10_ may be due to the relatively low PM_10_ levels in the Reykjavik area. Long-term exposure to high concentrations of PM_2.5_ may adversely affect cardiovascular health. Still, the potential adverse effect of coarse particles (PM_2.5-10_) should not be ignored. Unfortunately, we did not have access to PM_2.5_ measures and no information on the proportion of particles smaller than 2.5 μm in aerodynamic diameter within the PM_10_ concentrations was available.

We did not find any statistically significant associations of increases in H_2_S concentrations and *glyceryl trinitrate* dispensing at any time lag. However, introducing H_2_S into the 3-day mean analysis yielded the highest OR for NO_2_ and O_3_ at lag 0 and lag 1. So, H_2_S did not seem to be an independent risk factor for *glyceryl trinitrate* dispensing. The possible effect of H_2_S on cardiovascular morbidity has not been as intensively studied as NO_2_, PM_10_, and O_3_. Iceland offers a rare setting to study the association of H_2_S and morbidity and mortality based on the availability of detailed H_2_S measurements and population-based registries including individual health information. The source of H_2_S in Reykjavik is two geothermal power plants, Hellisheidarvirkjun and Nesjavallavirkjun, located 26 and 33 km east of the city, while the other pollutants in this study are mostly traffic-related. The few existing studies on H_2_S using mortality and hospital discharge as outcome measures suggest that long-term exposure to moderately high concentrations of H_2_S is associated with cardiovascular- and respiratory morbidity [33,34]. In Reykjavik, the intermittent concentrations of H_2_S are not likely to be evenly dispersed over the city, and therefore, these findings call for further analysis of the potential adverse health effects of H_2_S.

An imperative strength of this case-crossover study is the design that adjusts for seasonality, time trends, and slowly time-varying confounders, since the control exposures are within the same season and day of the week as the case exposure. The model makes within-subject comparisons possible and time-independent confounders are controlled for by design. Therefore, individual characteristic adjustments are not needed. In our assessments we controlled for temperature, relative humidity, and influenza season. Further, we tested whether different control samplings affected our results. This was not the case, as the pattern of associations between air pollutants and *glyceryl trinitrate* dispensing remained similar and statistically significant. Moreover, the Icelandic Medicines Registry is virtually complete, since all filled prescriptions in outpatient settings are registered according to personal identification numbers. Such completeness of a medication registry is quite unique. Finally, *glyceryl trinitrates* are not prescribed unless the patient has angina pectoris and therefore there is no incidence of dispensing the medication to individuals who do not suffer from cardiovascular disease.

The study has limitations that should be mentioned. Firstly, the data did not allow an identification of whether individuals to whom *glyceryl trinitrates* was dispensed were previously known to have angina pectoris, or if they were newly diagnosed with angina pectoris at the time of dispensing. Some patients with angina pectoris may of course have had *glyceryl trinitrates* at hand, ready to use in case of exacerbation, and thus do not need a new dispensation at the time of exacerbation, but may fill prescription at later time by way of precaution. Such course of events would diminish the possibility to find an association between drug dispensing and an increase in pollution concentrations with a short lag. Secondly, the air pollution data were only derived from one monitoring station. The study therefore has the inherent weakness that the exposure concentrations were not on an individual basis, but cover the whole population of the Reykjavik area with one station as a proxy for exposure. This monitoring station was the only one available in the Reykjavik area during the study period.

## Conclusions

To our knowledge, this is the first study where the dispensing of *glyceryl trinitrates* is used as a public health indicator in relation to ambient air pollution. Our results suggest that NO_2_ and O_3_ ambient air concentrations may adversely affect cardiovascular health, as measured by the dispensing of the anti-angina pectoris medication, *glyceryl trinitrates*.

## Abbreviations

CH4: Methane; CI: Confidence interval; CO: Carbon oxide; H2S: Hydrogen sulfide; NO2: Nitrogen dioxide; O3: Ozone; OR: Odds ratio; PM10: Particulate matter ≤10 μm in aerodynamic diameter; PM2.5: Particulate matter ≤2.5 μm in aerodynamic diameter; SD: Standard deviation; SO2: Sulfur dioxide.

## Competing interest

The authors have not over the past three years had any financial relations with organizations that might have an interest in the submitted work. The authors hereby declare no relationships or activities that could appear to have influenced the submitted work.

## Authors’ contributions

RGF, HZ, VR, and OO designed the study. RGF, VR, and OO planned the analysis, OO, RGF, and TT analyzed the data, RGF, HZ, and VR drafted the article, all authors read the manuscript, interpreted the conclusions, and agreed on the final version.
